# Autism-like behaviors in male mice with a *Pcdh19* deletion

**DOI:** 10.1186/s13041-019-0519-3

**Published:** 2019-11-20

**Authors:** Jisoo Lim, Jiin Ryu, Shinwon Kang, Hyun Jong Noh, Chul Hoon Kim

**Affiliations:** 1 Department of Pharmacology, BK21 PLUS Project for Medical Science, Brain Research Institute, Yonsei University College of Medicine, Seoul, 03722 Korea; 2 Severance Biomedical Science Institute, Yonsei University College of Medicine, Seoul, 03722 Korea

## Abstract

Mutations in protocadherin 19 (*PCDH19*), which is on the X-chromosome, cause the brain disease Epilepsy in Females with Mental Retardation (EFMR). EFMR is also often associated with autism-like symptoms. In mice and humans, epilepsy occurs only in heterozygous females who have a mixture of *PCDH19* wild-type (WT) and mutant cells caused by random X-inactivation; it does not occur in hemizygous *PCDH19* mutant males. This unique inheritance pattern strongly suggests the underlying disease mechanism operates via interference between WT and mutant cells rather than being a result of complete loss of PCDH19 functions. Although it remains unclear whether the other symptoms of EFMR also conform to this unique genotype-phenotype relationship, *PCDH19* mutant males were recently reported to demonstrate autism-like symptoms. We, therefore, used a *Pcdh19* knockout (KO) mouse model to ask whether a complete lack of PCDH19 causes autism-like behaviors. Consistent with the autism observed in EFMR females, we found *Pcdh19* heterozygous KO female mice (with mosaic expression of PCDH19) show defects in sociability in the 3-chamber test. Surprisingly, hemizygous *Pcdh19* KO male mice (without any PCDH19 expression) exhibit impaired sociability in the 3-chamber test and reduced social interactions in the reciprocal social interaction test. We also observed that, compared to WT mice, mutant mice display more repetitive behaviors, including self-grooming and rearing. These findings indicate that hemizygous *Pcdh19* KO male mice show autism-like phenotypes.

Epilepsy in Females with Mental Retardation (EFMR) is reportedly caused by mutations (i.e., missense, nonsense, and deletion, etc.) in the X-linked gene protocadherin 19 (*PCDH19*) [1]. EFMR patients have early-onset seizures frequently associated with varying degrees of intellectual disability (ID) and autism-like symptoms [2–4]. As the name of the disease implies, it is highly sex-limited. Epileptic symptoms appear only in females heterozygous for *PCDH19* mutations, whereas males hemizygous for *PCDH19* mutations are unaffected carriers [5]. Because *PCDH19* is X-linked, it is subject to random X-inactivation, producing mosaic expression in heterozygous mutant females [1]. The identification of male patients affected by postzygotic somatic *PCDH19* mutations supports the idea that the disease mechanism is related to mosaic expression of PCDH19 [6, 7]. This idea is further supported by the case of an epileptic patient with Klinefelter Syndrome (47, XXY) heterozygous for a *PCDH19* mutation [8]. This unique genotype-phenotype relationship suggests the symptoms of EFMR emerge from the abnormal interaction of two different populations of brain cells, some with and some without PCDH19 expression. This cellular mechanism is referred to as “cellular interference” [1, 6].

Recently, Pederick et al. [9] demonstrated a dramatic and abnormal segregation of PCDH19(+) and PCDH19(−) cells in the developing brains of *Pcdh19* heterozygous KO female mice that was well-correlated with seizure-like activities as recorded by electrocorticogram. This result provided the first experimental evidence of cellular interference as a key pathogenic mechanism in EFMR. It is unclear, however, whether autism spectrum disorder (ASD) in EFMR also conforms to both this unusual inheritance pattern and cellular interference mechanism. It is notable that recent human studies identified some males with ASD who have mutations in *PCDH19* [3, 10, 11], suggesting *PCDH19* mutations may also play a role in producing the symptoms of males with ASD via mechanisms other than cellular interference. In this study, we investigate this possibility using a *Pcdh19* KO mouse model.

Consistent with previous studies [9, 12], heterozygous *Pcdh19* KO female mice show an abnormal “tiger-striped” pattern of segregation between PCDH19(+) and PCDH19(−) cells in the brain (Fig. 1a). In contrast to heterozygous *Pcdh19* KO female mice, we did not observe a similar segregation in hemizygous *Pcdh19* KO male mice, despite both having a *tdTomato* (*tdT*) reporter gene on the X-chromosome where the *Pcdh19* gene was deleted (Fig. 1a). This finding suggests any phenotypes in hemizygous *Pcdh19* KO male mice are independent of the abnormal sorting mechanism observed in heterozygous *Pcdh19* KO female mice.

**Fig. 1:**
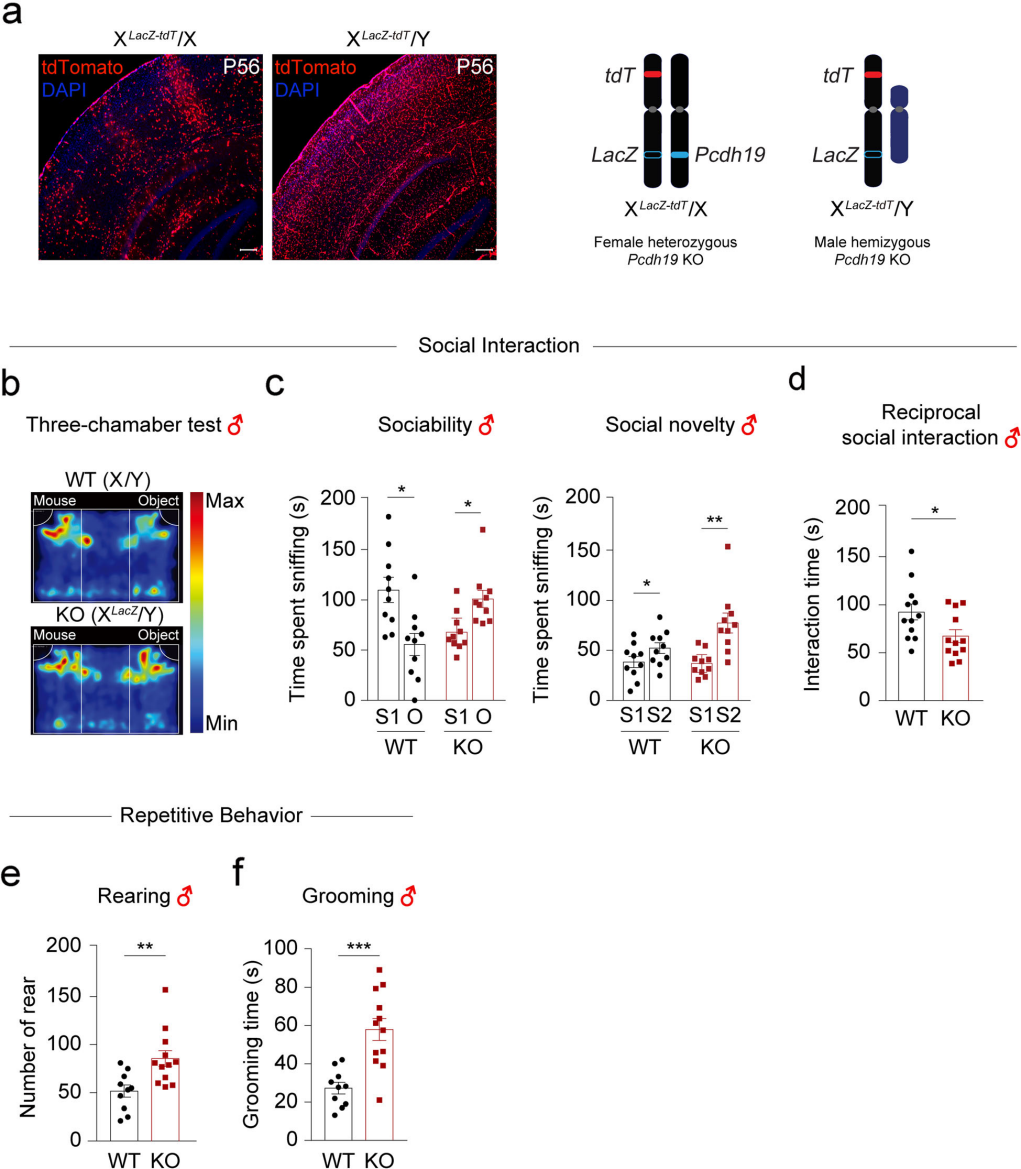
*Pcdh19* hemizygous KO male mice exhibit autism-like behaviors. **a** Representative images of *tdT*-expressing cells (PCDH19-negative cells) in coronal sections of HET-*tdT* female and KO-*tdT* male brains at P56 (left). Scale bar, 200 μm. Schematic diagrams of the X and Y chromosomes of heterozygous KO female and hemizygous KO male mice, showing *tdT* and *Pcdh19*-null alleles (in which the PCDH19 coding sequence is replaced with a *LacZ* cassette) are located on the same X-chromosome; X^*LacZ-tdT*^ (right). **b** Group-averaged heat map images for the movement of WT (X/Y) and *Pcdh19* hemizygous KO (X^*LacZ*^/Y) male mice during the 3-chamber sociability test (S1 vs O). **c** Quantification of the results shown as sniffing time, based on the time spent sniffing S1 vs O in the sociability test and S1 vs S2 in the social novelty test (*p < 0.05, **p < 0.01, paired Student’s *t* test). **d** The time of social interaction of *Pcdh19* KO male mice during the reciprocal social interaction test. **e-f** Repetitive behavior tests: the number of rearing incident (**e**) and the time spent self-grooming (**f**) in *Pcdh19* KO male mice (*p < 0.05, **p < 0.01, ***p < 0.001, unpaired Student’s *t* test). *n* = 10–12 male mice per genotype. All data are presented as means ± SEM

First, we asked whether heterozygous *Pcdh19* KO female mice show autism-like behaviors in our experimental setting (see Additional file 1 for the detailed methods). We found that heterozygous *Pcdh19* KO female mice (X^*LacZ*^/X) do not show any preference toward exploring a novel mouse (S1) versus a non-social novel object (O) in the 3-chamber test (the sociability test). This suggests heterozygous *Pcdh19* KO female mice recapitulate the autism-like symptoms of female EFMR patients. In the social novelty test, in which we measured preference toward a familiar mouse (S1) and a novel mouse (S2), we found the heterozygous *Pcdh19* KO mice (X^*LacZ*^/X) spend more time exploring the novel mouse (S2) (see Additional file 2).

We next examined the male mice to determine whether they also show any social impairment. In the 3-chamber sociability test, we found male *Pcdh19* KO (X^*LacZ*^/Y) mice spend significantly more time sniffing a non-social novel object (O) rather than a novel mouse (S1), suggesting abnormal sociality. Both hemizygous males and controls, however, show similar preference towards a novel mouse (S2) in the social novelty test (Fig. 1b–c). We then measured reciprocal social interactions to confirm the social abnormalities of male *Pcdh19* KO (X^*LacZ*^/Y) mice. We found they spend significantly less time interacting with a stranger mouse than WT (X/Y) mice do (Fig. 1d). To determine whether male *Pcdh19* KO (X^*LacZ*^/Y) mice show increased repetitive behavior—another autism-like phenotype—we monitored their rearing and stereotyped grooming behaviors. We found *Pcdh19* X^*LacZ*^/Y male mice spend more time rearing and self-grooming (Fig. 1e–f) than WT (X/Y) male mice. Thus, their abnormal social interaction results and increased repetitive behaviors suggest hemizygous *Pcdh19* KO (X^*LacZ*^/Y) mice show autism-like behaviors.

We do not yet know how the complete loss of *Pcdh19* causes autism-like behaviors in male mice, but considering the fact that the abnormal segregation pattern occurs only in the brain of female heterozygous *Pcdh19* KO mice [9, 13], but not in male KO mice, our present findings suggest the mechanism will be distinct from the cellular interference mechanism that underlies the epileptic symptoms of EFMR. It is possible a loss of PCDH19-mediated cell-to-cell adhesion may contribute to autism-like behaviors in hemizygous *Pcdh19* male KO mice. PCDH19 regulates intracellular binding proteins like NONO and the GABA_A_ receptor alpha subunits [14, 15]. Hence, it is also possible the absence of PCDH19 disrupts the function of unidentified autism-related binding proteins. Our social interaction results were inconsistent with a previous study in which heterozygous KO female and hemizygous KO male mice showed no abnormalities in the social interaction tests [12]. In fact, we are still unclear why the mice showed this inconsistency, but we found that the background of the mouse, the targeted exons, and the size of the behavioral apparatus used for the experiment were all different.

This is the first report showing, in genetically modified mice, that autism-like behaviors induced by *Pcdh19* mutations are not subject to the same genotype-phenotype relationship observed in epileptic symptoms of EFMR. From this finding, we postulate that both mosaic expression of *PCDH19* and *PCDH19* insufficiency contribute to the pathogenesis of EFMR. Considering the fact that male patients affected by mosaic *PCDH19* mutations also show autism [6], the induction of autism in a male patient may not require complete loss of PCDH19 in every cell. In the future, the generation of male mice with mosaic *Pcdh19* deletions will help address the question of whether both mosaic loss and complete loss of *PCDH19* result in autism-like behaviors.

## Supplementary information

**Additional file 1.** Materials and Methods.

**Additional file 2: Figure S1.** *Pcdh19* heterozygous KO female mice display autistic-like behaviors in the 3-chamber sociability test.

## Data Availability

All data and materials are available upon requests.

## Abbreviations

ASD: Autism spectrum disorder
EFMR: Epilepsy in females with mental retardation
ID: Intellectual disability
KO: Knockout
PCDH19: Protocadherin 19
SEM: Standard error of the mean
tdT: tdTomato
WT: Wild-type
