# Supplementary material for: Autism-like behaviors in male mice with a Pcdh19 deletion
Source: Mol Brain. 2019 Nov 20;12:95. doi: 10.1186/s13041-019-0519-3 (PMC6864969; doi:10.1186/s13041-019-0519-3)
Supplement: Supplementary file 1 — Additional file 1. Materials and Methods. [file 13041_2019_519_MOESM1_ESM.docx]

**Animals**

We purchased the B6;129SvEv-Pcdh19tm1 (TF2108) mouse from Taconic Biosciences. The LacZ cassette is inserted to replace *Pcdh19* exons 1–3. The resulting mice were then back-crossed with the C57BL/6J line. For fluorescence imaging, *Pcdh19*-*tdTomato* (*tdT*) mice (X*^LacZ-tdT^*/X and X*^LacZ-tdT^*/Y) were generated by crossing *Pcdh19* KO mice with mice carrying a CMV-Cre*;* CAG promoter/loxP-stop-loxP/tdTomato knock-in at the Hprt locus (CMV-Cre*;*Hprt-LSL-tdT) [1]. During this mating, a random X-chromosomal recombination between *Pcdh19* gene (133.6Mb) and *tdT* (53Mb) occurred, creating *Pcdh19-tdT* mice, which are both *Pcdh19*-null (replaced by the *LacZ* cassette) and positive for the *tdT* gene on the same X-chromosome.

For the behavioral analyses, WT (X/Y) male and *Pcdh19* heterozygous KO (HET; X*^LacZ^*/X) female mice were crossed to obtain WT (X/Y) and *Pcdh19* hemizygous KO (X*^LacZ^*/Y) male littermates as well as WT (X/X) and HET (X*^LacZ^*/X) female littermates. The offspring were genotyped via PCR using three primers: P1, F-5′-CGAGTCCACTACCGACTCTGCT-3′, for both the WT and *Pcdh19*-null alleles; P2, R-5′-AGCCCGGCTACTCAGTTTTCC-3′, for the *Pcdh19* WT allele; and P3, R-5′- CTGCAAAGGGTCGCTACAGACG-3′, for the *Pcdh19*-null allele. The expected WT and null allele products were 130 bp and 380 bp, respectively. All mice were housed in groups with *ad libitum* access to food and water in a humidity- and temperature-controlled, specific pathogen-free environment (12-h light cycle; lights on at 8 am) in the Yonsei University College of Medicine Animal Care Facility. Animal cages were changed by the experimenters once a week. Male mice were 4–7-months old (*n* = 10–12 per genotype) at the onset of behavioral testing. Behavioral experiments were performed in the following order: 3-chamber test, reciprocal social interaction test, and measurement of repetitive behaviors (10-min video recording to measure both self-grooming time and the number of rearing events). Female mice were only tested in the 3-chamber test at the age of 5–9 months (*n* = 8–10 per genotype). All animals were handled daily for a week prior to behavioral testing. All animal experiments were approved (No. 2018-0285) by the Animal Care Committee of Yonsei University College of Medicine using the US National Institutes of Health Guidelines.

**Fluorescence imaging**

Mice were anesthetized and perfused with 4% paraformaldehyde (wt/vol) in 0.1M PBS (pH 7.4), post-fixed in the same solution for overnight at 4℃. The brain samples were cut into 50 μm coronal sections using a vibratome (VT1200S, Leica Biosystems, Germany). Sections were stained with DAPI (1:2,000) for 1 h, washed three times and mounted with Flouromount-G (OB100-01, SouthernBiotech, Birmingham, AL, USA) and observed under Axio Image M2 (Carl Zeiss, Jena, Germany). Coronal sections used in this study is -3.08 mm relative to bregma of the mouse brain.

**Three-chamber test**

Mice were adapted to the test room for at least for 1 h before the test. The 3-chamber sociability and social novelty tests were performed as described previously [2,3], with minor modifications. The test apparatus was a transparent Plexiglas box (60 W × 40 D × 20 H cm) divided into left, centre, and right chambers with two transparent partitions with square opening doors (8 x 6 cm). For habituation, a test mouse was allowed to explore the entire environment for 10 min with the doors opened. An empty cylindrical wire-cage (10 cm in diameter) was used as an inanimate object stimulus (O) and an identical cylindrical wire-cage containing a novel mouse was used as the social stimulus (S1). A test mouse was placed in the centre chamber with the doors closed, and both O and S1 were introduced to the top corner of each side chamber. Upon opening the doors, mice behavior was recorded with a video camera for 10 min to test sociability (S1 vs O). For the social novelty test, the test mouse was again placed in the centre with the doors closed and the process was repeated except with another novel mouse (S2) instead of the inanimate object stimulus$-$a test mouse was allowed to freely explore both the S1 (familiar) and S2 (unfamiliar) mice for another 10 min. Between each subject, the arena and cylindrical wire-cages were cleaned with 70% ethanol and wiped with dry paper. Novel sex- and age-matched mice from the FVB/J background were carefully chosen and never exposed to the test mouse until testing began. Novel mice were habituated to the wire cage for 10 min prior to the test session. The time spent in sniffing each target were quantified using the EthoVision XT 14 software package (Noldus) and group-averaged heat map images for each genotype were generated in the process.

**Reciprocal social interaction test**

Mice were adapted to the test room for at least 1 h prior to behavioral testing. A test mouse was placed in a clean mouse cage (39.1 W x 19.9 D x 16 H cm) with an unfamiliar mouse from the same background, sex, and genotype and video-recorded for 10 min. The time the test mice engaged in all types of social interactions, including any physical contact, nose-to-nose sniffing, nose-to-anus sniffing, following, and crawling on or under each other was manually scored by two independent observers with assistance from the EthoVision XT 14 software package (Noldus). The observers were blind to the genotype of the mice and the inter-observer reliability was assessed (percent agreement is 0.91).

**Repetitive self-grooming and rearing**

Male mice were adapted to the test room for 30 min prior to video recording. Each mouse was placed individually into a new mouse cage with scented bedding materials from their home cage and habituated for 5 min. Mice behavior was video-recorded for 10 min within the time window from 10 am to 2 pm. A video camera was mounted approximately 50 cm in front of the test arena to gain a lateral view, and the observer was absent during the 10 min recording session so as to minimize distractions. The self-grooming duration was manually scored, and the number of rearing events was automatically scored using the EthoVision XT 14 software package (Noldus). The number of rearing events was automatically counted when the nose-point entered the zone above which each mouse could reach by raising its forelimbs from the ground.

**Statistical analysis**

Data analysis was conducted with GraphPad Prism v. 7 (GraphPad Software, Inc., La Jolla, CA, USA). Two-tailed paired Student’s *t* tests were used to compare time spent sniffing in each target (S1 vs O or S2) in the 3-chamber tests within each genotype. Otherwise, two-tailed unpaired Student’s *t* tests were used to compare the mean differences between the two genotypes. Probability values less than 0.05 were considered statistically significant. All data are presented as means ± SEM.

**References**

1. Wu H, Luo J, Yu H, Rattner A, Mo A, Wang Y, et al. Cellular resolution maps of X chromosome inactivation: implications for neural development, function, and disease. Neuron. 2014;81(1):103-19.

2. Moy SS, Nadler JJ, Perez A, Barbaro RP, Johns JM, Magnuson TR, et al. Sociability and preference for social novelty in five inbred strains: an approach to assess autistic-like behavior in mice. Genes Brain Behav. 2004;3(5):287-302.

3. Silverman JL, Tolu SS, Barkan CL, Crawley JN. Repetitive self-grooming behavior in the BTBR mouse model of autism is blocked by the mGluR5 antagonist MPEP. Neuropsychopharmacology. 2010;35(4):976-89.
