# Supplementary material for: Autism-like behaviors in male mice with a Pcdh19 deletion
Source: Mol Brain. 2019 Nov 20;12:95. doi: 10.1186/s13041-019-0519-3 (PMC6864969; doi:10.1186/s13041-019-0519-3)
Supplement: Supplementary file 2 — Additional file 2: Figure S1. Pcdh19 heterozygous KO female mice display autistic-like behaviors in the 3-chamber sociability test. [file 13041_2019_519_MOESM2_ESM.docx]

**Figure S1.** *Pcdh19* heterozygous KO female mice display autistic-like behaviors in the 3-chamber sociability test. **a** Group averaged heat map images for the movement of WT (X/X) and *Pcdh19* heterozygous KO (HET; X*^LacZ^*/ X) female mice during the 3-chamber sociability test (S1 vs O). **b** Quantification of the results shown as time spent sniffing of S1 vs O in the sociability test and S1 vs S2 in the social novelty test of WT and *Pcdh19* HET female mice (**p* < 0.05, ***p* < 0.01, paired Student’s *t* test). *n*=8-10 for female mice per genotype. All data are presented as means ± SEM.
